# Effects of the novel selective κ-opioid receptor agonist NP-5497-KA on morphine-induced reward-related behaviors

**DOI:** 10.1038/s41598-023-45584-4

**Published:** 2023-10-24

**Authors:** Soichiro Ide, Toshitake Hirai, Takafumi Muto, Tomio Yamakawa, Kazutaka Ikeda

**Affiliations:** 1 Addictive Substance Project, Tokyo Metropolitan Institute of Medical Science, 2-1-6 Kamikitazawa, Setagaya-ku, Tokyo, 156-8506 Japan; 2 Discovery Research Laboratories, Nippon Chemiphar Co., Ltd., 1-22 Hikokawado, Misato, Saitama 341-0005 Japan

**Keywords:** Pharmacology, Addiction

## Abstract

Opioid addiction and the opioid overdose epidemic are becoming more serious, and the development of therapeutic agents is essential for the pharmacological treatment of substance use disorders. The κ-opioid receptor (KOP) is a member of the opioid receptor system that has been gaining attention as a promising molecular target for the treatment of numerous human disorders, including pain, depression, anxiety, and drug addiction. Here, we biologically and pharmacologically evaluated a novel azepane-derived ligand, NP-5497-KA, as a selective KOP agonist. NP-5497-KA had 1000-fold higher selectivity for the KOP over the μ-opioid receptor (MOP), which was higher than nalfurafine (KOP/MOP: 65-fold), and acted as a selective KOP full agonist in the 3′,5′-cyclic adenosine monophosphate assay. The oral administration of NP-5497-KA (1–10 mg/kg) dose-dependently suppressed morphine-induced conditioned place preference in C57BL/6 J mice, and its effects were comparable to an intraperitoneal injection of nalfurafine (1–10 μg/kg). Nalfurafine (10 μg/kg) significantly inhibited rotarod performance, whereas NP-5497-KA (10 mg/kg) exerted no effect on rotarod performance. These results indicate that NP-5497-KA may be a novel option for the treatment of opioid use disorder with fewer side effects.

## Introduction

Opioids exert their pharmacological effects by acting on μ (MOP), κ (KOP), and δ (DOP) opioid receptors. Morphine, which exhibits high affinity for MOPs, has long been used as an analgesic. However, morphine and other MOP agonists are known to cause adverse events, such as nausea, vomiting, constipation, respiratory depression, addiction, and tolerance, via MOPs^1,2^. KOP agonists also exert analgesic effects and are known to not induce adverse events that are seen with morphine. However, KOP agonists are generally known to have other side effects, such as psychotomimetic effects, dysphoria, and sedation^3,4^. Although various compounds have been reported to be KOP agonists, none of these compounds show selectivity for the KOP, high activity at the KOP, excellent in vivo stability, and no sedative or aversive drug effects. Nalfurafine (also known as TRK-820) is a recently developed moderately selective KOP agonist that has high activity at the KOP, excellent in vivo stability, and no sedative or aversive drug effects^5^. Nalfurafine has been used in Japan for the treatment of pruritus in patients who undergo hemodialysis and patients with chronic liver diseases^6–8^. It is not used as an analgesic agent because it exerts sedative effects at analgesic doses.

Especially in the United States, the problem of opioid addiction and the opioid-overdose epidemic, also known as the “opioid crisis,” is becoming more serious. The development of therapeutic agents is essential for the pharmacological treatment of substance use disorders, including opioid use disorder, and is being researched and developed worldwide. Addictive drugs are well known to increase extracellular levels of dopamine in the nucleus accumbens. In the nucleus accumbens, KOP agonist administration has been shown to decrease extracellular dopamine concentrations through the inhibition of dopamine release and by upregulating dopamine transporter reuptake activity^9,10^. U-50,488H, a selective KOP agonist, suppresses morphine-induced conditioned place preference (CPP) in mice^11^. It was also reported to attenuate cocaine-induced increases in extracellular dopamine in the nucleus accumbens in rats^12^. Furthermore, U-69,593, another selective KOP agonist, was reported to decrease cocaine self-administration in rhesus monkeys^13^. Therefore, novel KOP agonists may play a role in reducing significant morbidity and mortality that are associated with drug addiction. Our recent patent reported a new series of structurally novel compounds that potently activate the KOP. Among these compounds was NP-5497-KA, a novel azepane-derived ligand with oral bioavailability, which has a completely different structure from classic KOP agonists. The present study determined the selectivity of NP-5497-KA for each opioid receptor subtype and its agonistic activity. Conditioned place preference tests were conducted to test the inhibitory effect of NP-5497-KA on morphine preference compared with the clinically used nalfurafine.

## Materials and methods

### In vitro studies

#### Drugs

For the in vitro assays, NP-5497-KA, nalfurafine hydrochloride, the MOP-selective agonist DAMGO, the KOP-selective agonist U-69,593 (Sigma Aldrich, St. Louis, MO, USA), and the DOP agonist SNC80 (Toronto Research Chemicals, Toronto, ON, Canada) were dissolved in dimethylsulfoxide. NP-5497-KA and nalfurafine were synthesized by Nippon Chemiphar according to patent-related information.

#### 3′,5′-Cyclic adenosine monophosphate (cAMP) assay

The cAMP assay was performed using a homogeneous time-resolved fluorescence resonance energy transfer (TR-FRET) immunoassay and the LANCE *Ultra* cAMP kit (PerkinElmer, Waltham, MA, USA). Chinese hamster ovary K1 (CHO-K1) cells that stably expressed human opioid receptors were purchased from ChanTest (Cleveland, OH, USA). Thawed cells were resuspended in cAMP assay buffer (Hank’s Balanced Salt Solution, 5 mM HEPES, 0.5 mM isobutyl methylxanthine, and 0.1% bovine serum albumin, pH 7.4) with 2 × 10^5^ cells/ml for MOP- and DOP-expressing cells and 1 × 10^5^ cells/ml for KOP-expressing cells to match the stimulatory response induced by forskolin. The same volume (5 μl) of the cell suspension was added to the test compounds with 10 μM forskolin. The agonist assay was performed for NP-5497-KA and nalfurafine in the concentration range of 10^−7^ to 10^−14^ M for KOP and 10^−5^ to 10^−12^ M for MOP and DOP and for the reference compounds in the concentration range of 10^−5^ to 10^−12^ M. The full agonists SNC80, DAMGO, and U-69,593 were used as the reference compounds (positive controls) for DOP-, MOP-, and KOP-induced cAMP inhibition, respectively. The cells were stimulated for 30 min at room temperature (25 °C), and cAMP measurements were performed. A total of 5 μl of Eu cAMP Tracer Working Solution and 5 μl of U*Light*-anti-cAMP Tracer Working Solution were added, mixed, and incubated for 1 h. The TR-FRET signal was read on an EnVision microplate reader (PerkinElmer, Waltham, MA, USA). Data were calculated with a dose–response curve (EC_50_ and E_max_) using Prism 7 software (GraphPad, San Diego, CA, USA).

### In vivo studies

#### Animals

Male ICR mice (Japan SLC, Shizuoka, Japan) were used at 5–6 weeks of age (22–34 g) for the rotarod tests and supplemental pharmacokinetic study. The total number of mice for these experiments was 69, of which 47 were used for supplemental experiments. Male C57BL/6 J mice (Japan CLEA, Tokyo, Japan) were used at 7 weeks of age (21–25 g) for the other behavioral experiments. The total number of mice for these experiments was 114, of which 54 were used for supplemental experiments. All behavioral experiments examined only a single dose for each mouse and were not repeated. The mice were housed 4–6 per cage in an environment at 23 ± 1 °C and 50 ± 5% humidity with free access to food and water under a 12 h/12 h light/dark cycle. All experiments were performed with approval from the Institutional Animal Care and Use Committee at the Tokyo Metropolitan Institute of Medical Science (22–012) and Nippon Chemiphar Co., Ltd. (18–23, 18–32 and 19–30), and all experiments were performed in accordance with relevant guidelines and regulations.

#### Drugs for pharmacological study

Morphine hydrochloride (Takeda Pharma, Osaka, Japan) was dissolved and diluted in saline and administered intraperitoneally (i.p.) in a volume of 10 ml/kg body weight. NP-5497-KA and nalfurafine hydrochloride were dissolved and diluted in distilled water and administered in a volume of 10 ml/kg body weight.

#### Conditioned place preference test

The CPP test was performed using a two-compartment Plexiglas chamber like as our previous report ^14^ with slight modifications. One compartment (175 mm width × 150 mm length × 175 mm height) had a black floor and walls with an equally spaced stainless-steel stripe-like grid on the floor. The other compartment had the same dimensions but had a white floor and walls with a stainless-steel grid on the floor. For the preconditioning and postconditioning phases, a T-style division with double 60 mm × 60 mm openings allowed the mice to access both compartments. During the conditioning phases, the openings were closed to restrict the mice to one of the compartments. The CPP apparatus was placed in a sound-attenuated, light-controlled box. On day 1 (preconditioning: habituation) and day 2 (preconditioning: pretest), the mice were allowed to freely explore both compartments for 900 s, and the time spent in each compartment during the exploratory period and locomotor activity were measured by infrared detectors (Neuroscience, Osaka, Japan). We selected a counterbalanced protocol to nullify each mouse’s initial preference as discussed previously^15^. Conditioning was conducted once daily for 4 consecutive days (days 3–6). The mice were injected with morphine (10 mg/kg, i.p.) or saline and immediately confined to the black or white compartment for 60 min on day 3. On day 4, the mice were injected with alternate saline or drug injections and immediately confined to the opposite compartment for 60 min. On days 5 and 6, the same conditioning as on days 3 and 4 was repeated. The assignment of the mice to the conditioned compartment was performed randomly and counterbalanced across subjects. During the postconditioning phase on day 7, the time spent in each compartment was measured for 900 s. The CPP score was designated as the time spent in the drug-paired compartment on day 7 minus the time spent in the same compartment in the preconditioning phase on day 2. The effects of NP-5497-KA and nalfurafine on the rewarding properties of morphine were tested in mice that received oral or intraperitoneal administration of either NP-5497-KA (1, 3, and 10 mg/kg) or nalfurafine (1, 3, and 10 μg/kg) 30 or 5 min before each morphine injection, respectively.

#### Locomotor activity

Locomotor activity was assessed with an animal activity-monitoring apparatus that was equipped with infrared detectors (SUPERMEX, CompACT FSS, Muromachi Kikai Co., Tokyo, Japan). The mice were placed individually in 30 cm × 45 cm × 30 cm plastic cages to which they had not been previously exposed under dim light and sound-attenuated conditions. Locomotor activity was first monitored for 40 min, and then 10 mg/kg morphine was administered (i.p.), and the animals were monitored for morphine-induced effects for 60 min. The effects of NP-5497-KA and nalfurafine on morphine-induced hyperlocomotion were tested in mice that received oral or intraperitoneal administration of either NP-5497-KA (10 mg/kg) or nalfurafine (10 μg/kg) 30 or 5 min before each morphine injection, respectively.

#### Rotarod test

Rotarod testing was performed using a rotarod apparatus (KN-75, Natsume Seisakusho Co., Ltd., Tokyo, Japan). Male ICR mice were pretrained to maintain their position on a rotarod (3 cm diameter) at 3 rotations per minute (rpm) for ≥ 60 s. Next, they were trained on the rotarod at 3, 4, and 5 rpm for 180 s. After training, the mice were placed on the rotarod at 5 rpm. The time for each mouse to fall from the rotarod was recorded. The cut-off time was set at 300 s. The effects of NP-5497-KA and nalfurafine on rotarod performance were tested in mice that received NP-5497-KA (10 mg/kg, p.o.) or nalfurafine (3 and 10 μg/kg, subcutaneous [s.c.]).

### Statistical analysis

All of the data were normally distributed and are expressed as the mean ± the standard error of the mean (SEM). The data were analyzed using one-way analysis of variance (ANOVA) followed by Sidak’s multiple-comparison post hoc test or Dunnett’s multiple-comparison post hoc test, two-way repeated-measures ANOVA followed by Sidak’s multiple-comparison post hoc test, or two-way ANOVA followed by Dunnett’s multiple-comparison post hoc test using Prism 7 software (GraphPad, La Jolla, CA, USA). Values of *p* < 0.05 were considered statistically significant.

### Ethics approval and consent to participate

All experiments were performed with approval from the Institutional Animal Care and Use Committee at the Tokyo Metropolitan Institute of Medical Science (22–012) and Nippon Chemiphar Co., Ltd. (18–23, 18–32 and 19–30), and all experiments were performed in accordance with relevant guidelines and regulations. This study is reported in accordance with ARRIVE guidelines.

## Results

### In vitro pharmacological study

**Figure 1:**
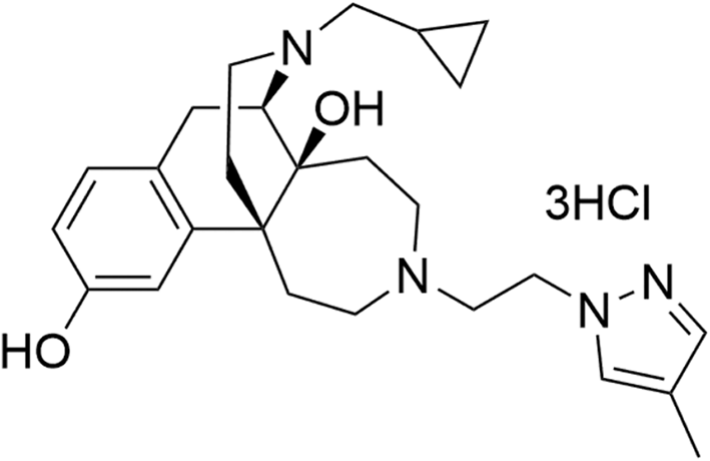
Chemical structure of NP-5497-KA.

To identify small molecules that activate KOP selectively, we initially screened a chemical library using the cAMP assay. NP-5497-KA (Fig. 1) selectively activated KOP-mediated cAMP inhibition. NP-5497-KA had high potency (EC_50_ = 0.014 nM) and high efficacy (E_max_ = 97%) at the KOP, and its potency was approximately 90-fold higher than the KOP agonist nalfurafine hydrochloride and 1600-fold higher than the KOP agonist U-69,593 (Table 1). NP-5497-KA showed approximately 100- and 1000-fold selectivity for the KOP over the DOP and MOP, respectively. For the MOP, both NP-5497-KA and nalfurafine acted as partial agonists, with maximal effects of 33% and 64%, respectively, of the full agonist DAMGO. For the DOP, NP-5497-KA acted as a full agonist and nalfurafine acted as a partial agonist, with maximal effects of 87% and 45%, respectively, of the full agonist SNC80.

**Table 1:** EC_50_ and E_max_ values of NP-5497-KA and Nalfurafine in the cAMP assay.

	KOP		MOP		DOP	
	*EC*_*50*_* (nmol/L)*	*E*_*max*_* (%)*	*EC*_*50*_* (nmol/L)*	*E*_*max*_* (%)*	*EC*_*50*_* (nmol/L)*	*E*_*max*_* (%)*
Compound	Geometric mean [95% CI] (lower–upper)	Mean ± SEM	Geometric mean [95% CI] (lower–upper)	Mean ± SEM	Geometric mean [95% CI] (lower–upper)	Mean ± SEM
Control	U-69,593		DAMGO		SNC80	
	27 [15–49]	100	5.9 [4.4–7.9]	100	5.6 [3.8–8.3]	100
NP-5497-KA	0.014 [0.010–0.021]	97 ± 1	14 [7.8–26]	33 ± 2	1.6 [1.2–2.1]	87 ± 1
Nalfurafine	0.12 [0.065–0.21]	91 ± 1	7.8 [7.1–8.5]	64 ± 1	132 [89–195]	45 ± 2

EC_50_ and E_max_ are expressed as the mean with the 95% CI or SEM from three independent experiments that were performed in duplicate. E_max_ is the maximal response of the test compound, expressed as a percentage of the maximal response to the DOP agonist SNC80, MOP agonist DAMGO, and KOP agonist U-69,593. CI, confidence interval.

In preparation for the in vivo pharmacological testing of NP-5497-KA, we sought to characterize preliminary pharmacokinetic features of this compound that could limit its utility in vivo or clinically. NP-5497-KA was metabolically stable when incubated with human and mouse hepatocytes (see Supplemental Methods, Supplementary Table S1). We further evaluated in the preliminary pharmacokinetic studies whether this compound had sufficient oral absorption. Following an oral dose of 10 mg/kg in mice, NP-5497-KA was rapidly absorbed, and peak plasma concentrations were observed 30 min after administration (see Supplemental Methods, Supplementary Table S2).

### Effects of NP-5497-KA and nalfurafine on morphine-induced CPP

**Figure 2:**
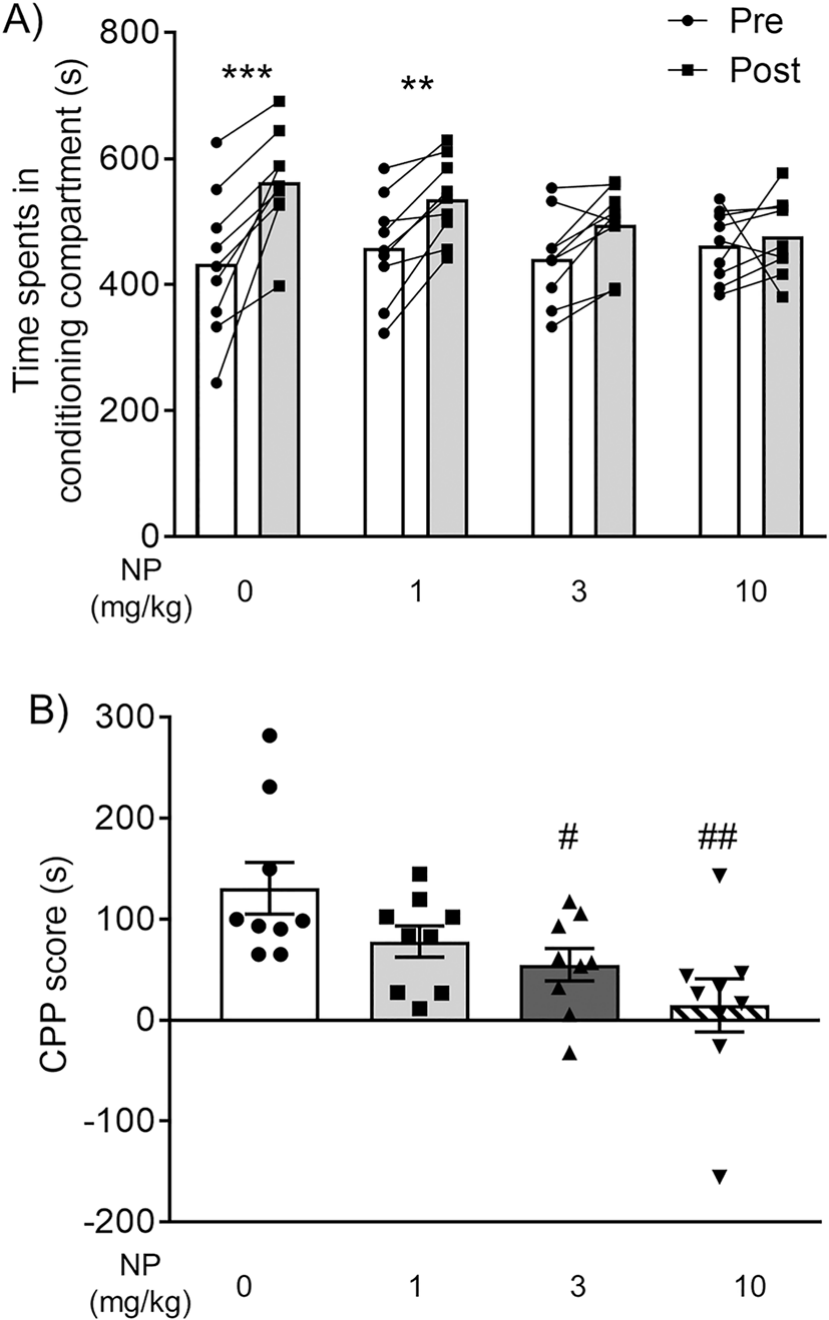
Inhibitory effect of NP-5497-KA (NP) on morphine-induced rewarding effects. (**A**) Time spent in the drug-paired compartment in the preconditioning (Pre, white columns) and postconditioning (Post, gray columns) phases. Mice were pretreated with NP-5497-KA (0, 1, 3, and 10 mg/kg, p.o.; *n* = 9/group) 30 min before each morphine treatment (10 mg/kg, i.p.) during the conditioning phase, respectively. The lines that connect symbols represent the value of each individual mouse, and the columns represent the mean. **p < 0.01, ***p < 0.001, difference between pre- and postconditioning phase in each treatment. (**B**) Conditioned place preference (CPP) scores for each treatment in mice. The columns and vertical lines represent the mean ± SEM. ^#^*p* < 0.05, ^##^*p* < 0.01, compared with vehicle-pretreated (control) mice.

To assess the inhibitory effect of NP-5497-KA on morphine-induced rewarding effects, we conducted the morphine-induced CPP test. Mice were pretreated with NP-5497-KA (0, 1, 3, and 10 mg/kg, p.o.) 30 min before each morphine injection during the conditioning phase. A two-way repeated-measures ANOVA revealed a significant NP dose × time spent interaction for morphine preference (*F*_3,32_ = 5.107,* p* = 0.0053). Post hoc tests revealed that morphine significantly increased the time spent in the previously paired compartment in vehicle-pretreated control mice (preconditioning: 432.8 ± 38.6 s; postconditioning: 563.3 ± 27.4 s; *p* < 0.001; Fig. 2A). There were no significant differences in the time spent in the previously paired compartment between the pre- and postconditioning phases in the 10 mg/kg NP-5497-KA-pretreated group (preconditioning: 461.8 ± 18.6 s; postconditioning: 476.4 ± 21.0 s) and 3 mg/kg NP-5497-KA-pretreated group (preconditioning: 435.8 ± 24.2 s; postconditioning: 495.5 ± 21.0 s; Fig. 2A), although there were significant differences in the time spent in the previously paired compartment between the pre- and postconditioning phases in the groups that were treated with 1 mg/kg NP-5497-KA (preconditioning: 457.9 ± 27.9 s; postconditioning: 535.8 ± 21.7 s; *p* = 0.004). The one-way ANOVA of CPP scores showed significant differences among the pretreated doses of NP-5497-KA before morphine treatment (*F*_3,32_ = 5.11,* p* = 0.005). Post hoc comparisons indicated that morphine-induced CPP scores were dose-dependently reduced in the NP-5497-KA-pretreated groups (1 mg/kg, *p* = 0.215; 3 mg/kg, *p* = 0.046; 10 mg/kg, *p* = 0.002; Dunnett’s multiple-comparison post hoc test; Fig. 2B) compared with the vehicle-pretreated group.

To compare the inhibitory effect of NP-5497-KA on morphine-induced rewarding effects with the other KOP agonist, we conducted the morphine-induced CPP test in mice that were pretreated with nalfurafine (0, 1, 3, and 10 µg/kg, i.p.) 5 min before each morphine injection during the conditioning phase. A two-way repeated-measures ANOVA revealed a significant nalfurafine dose × time spent interaction for morphine preference (*F*_3,26_ = 4.804,* p* = 0.0086). Post hoc tests revealed that morphine significantly increased the time spent in the previously paired compartment in vehicle-pretreated control mice (preconditioning: 465.2 ± 33.8 s; postconditioning: 560.3 ± 31.6 s; *p* < 0.001; Supplementary Fig. S1A). There were no significant differences in the time spent in the previously paired compartment between the pre- and postconditioning phases in the 3 μg/kg nalfurafine-pretreated group (preconditioning: 444.4 ± 43.5 s; postconditioning: 472.1 ± 27.9 s) and 10 µg/kg nalfurafine-pretreated group (preconditioning: 445.6 ± 36.5 s; postconditioning: 465.4 ± 30.8 s; Supplementary Fig. S1A). There were significant differences in the time spent in the previously paired compartment between the pre- and postconditioning phases in the 1 µg/kg nalfurafine-pretreated group (preconditioning: 450.0 ± 37.3 s; postconditioning: 509.7 ± 33.2 s; *p* = 0.005; Supplementary Fig. S1A). The one-way ANOVA of CPP scores showed significant differences among pretreated doses of nalfurafine before morphine treatment (*F*_3,26_ = 4.80,* p* = 0.009). Post hoc comparisons indicated that morphine-induced CPP scores were significantly and dose-dependently reduced in the nalfurafine-pretreated groups (1 µg/kg, *p* = 0.295; 3 µg/kg, *p* = 0.017; 10 µg/kg, *p* = 0.006; Dunnett’s multiple-comparison post hoc test; Supplementary Fig. S1B) compared with the vehicle-pretreated group.

### Effect of NP-5497-KA and nalfurafine on the expression of morphine-induced hyperlocomotion

**Figure 3:**
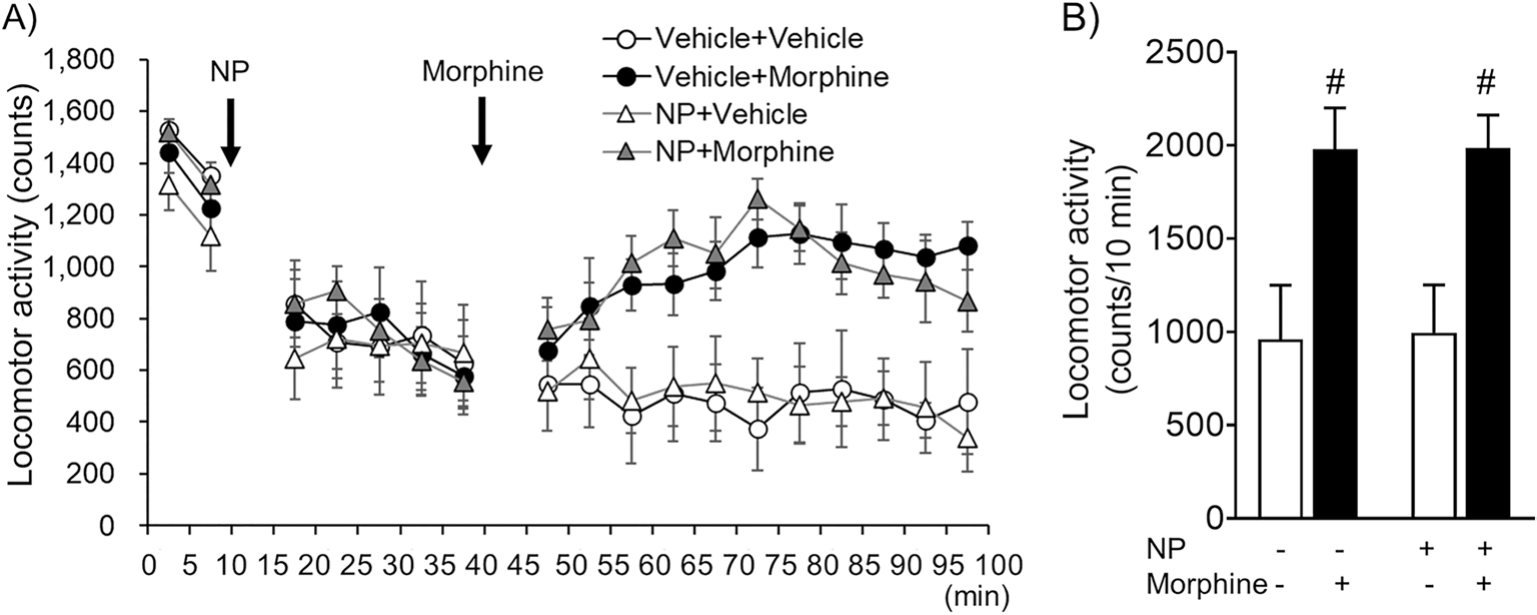
Effect of NP-5497-KA (NP) on morphine-induced hyperlocomotion. (**A**) Locomotor activity was measured before (40 min for habituation) and after (60 min) morphine treatment (10 mg/kg). (**A**) Mice (*n* = 6/group) were pretreated with NP-5497-KA (10 mg/kg, p.o.) or vehicle (p.o.) as indicated 30 min before morphine or saline treatment (i.p.). (**B**) Average number of locomotor counts in 10 min blocks after morphine administration for each treatment in mice. The columns and vertical lines represent the mean ± SEM. ^#^*p* < 0.05, compared with saline-treated mice (white column).

We assessed the effect of NP-5497-KA on the expression of morphine-induced hyperlocomotion (Fig. 3A,B). Pretreatment with 10 mg/kg NP-5497-KA (p.o.) alone did not affect locomotion in mice. Morphine (10 mg/kg, i.p.) significantly increased locomotion both with and without NP-5497-KA pretreatment (Fig. 3A). The two-way ANOVA of average locomotor activity after morphine treatment showed significant differences among groups (morphine effect: *F*_1,20_ = 17.58,* p* < 0.001; NP-5497-KA effect: *F*_1,20_ = 0.008,* p* = 0.932; morphine × NP-5497-KA interaction: *F*_1,20_ = 0.003,* p* = 0.956). Post hoc comparisons indicated that morphine significantly increased locomotion both with and without NP-5497-KA pretreatment (*p* = 0.049 and *p* = 0.041, respectively; Sidak’s multiple-comparison post hoc test; Fig. 3B) compared with each vehicle-treated control group.

The effect of nalfurafine on the expression of morphine-induced hyperlocomotion was also investigated (Supplementary Fig. S2A, B). Pretreatment with 10 µg/kg nalfurafine (i.p.) alone did not affect locomotion in mice. Morphine (10 mg/kg, i.p.) significantly increased locomotion both with and without nalfurafine pretreatment (Supplementary Fig. S2A). The two-way ANOVA of average locomotor activity after morphine treatment showed significant differences among groups (morphine effect: *F*_1,20_ = 17.20,* p* < 0.001; nalfurafine effect: *F*_1,20_ = 0.028,* p* = 0.869; morphine × nalfurafine interaction: *F*_1,20_ = 0.382,* p* = 0.544). Morphine administration increased locomotor activity in mice, but nalfurafine treatment did not appear to block the morphine-induced increase in locomotor activity (Supplementary Fig. S2B).

### Effect of NP-5497-KA and nalfurafine on rotarod performance

**Figure 4:**
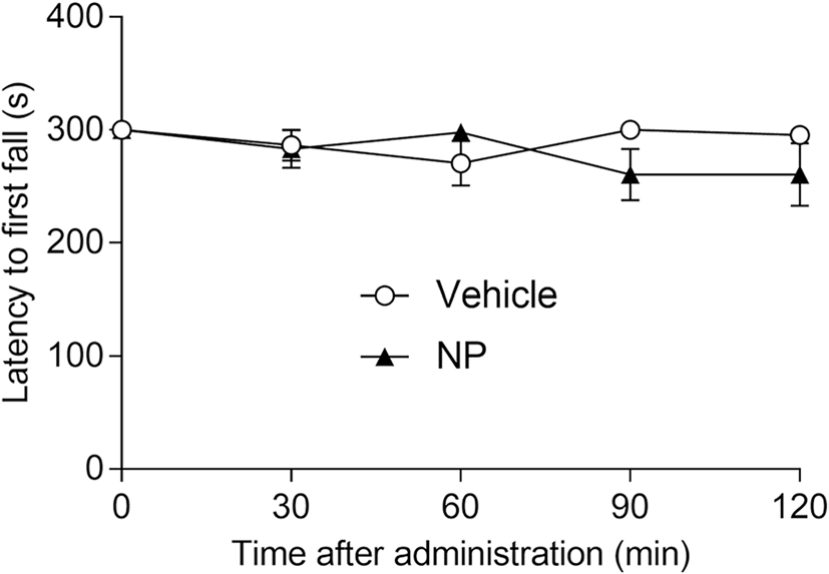
Effect of NP-5497-KA (NP) on rotarod performance. Rotarod performance was measured after the oral administration of NP-5497-KA (10 mg/kg). Mice (*n* = 11/group) were treated with NP-5497-KA (0 and 10 mg/kg, p.o.; black triangles) or vehicle (white circles). The data are expressed as the mean ± SEM.

Because KOP agonists produce substantial sedation, we further evaluated the effect of NP-5497-KA on rotarod performance in each 30 min period of the total 120 min session (Fig. 4). NP-5497-KA (10 mg/kg, p.o.) did not affect rotarod performance in mice (two-way ANOVA; no effect of NP-5497-KA treatment: *F*_1,20_ = 1.456, *p* = 0.242; no effect of time: *F*_4,80_ = 0.645, *p* = 0.632; no NP-5497-KA treatment × time interaction: *F*_4,80_ = 1.640, *p* = 0.172).

We next investigated the effect of nalfurafine on rotarod performance (Supplementary Fig. S3). Nalfurafine (10 µg/kg, s.c.) significantly inhibited rotarod performance. The two-way ANOVA of average rotarod performance after nalfurafine treatment showed significant differences among groups (effect of nalfurafine treatment: *F*_2,29_ = 8.006, *p* = 0.002; effect of time: *F*_4,116_ = 6.330, *p* = 0.0001; nalfurafine treatment × time interaction: *F*_8,116_ = 3.144, *p* = 0.003). Dunnett’s multiple-comparison post hoc test indicated that 10 µg/kg nalfurafine significantly inhibited rotarod performance 30 and 60 min after nalfurafine treatment (30 min, *p* < 0.0001; 60 min, *p* = 0.012) compared with the vehicle-treated control group.

## Discussion

The present study found that NP-5497-KA, a newly synthesized KOP agonist, had higher potency in activating the KOP and also higher selectivity for the KOP over the MOP (1,000-fold) than nalfurafine (KOP/MOP: 65-fold). The KOP/MOP selectivity of nalfurafine in the cAMP assay in the present study was high compared with previous studies that used binding experiments and guanosine 5′-*O*-(γ-thio)triphosphate assays (2.4–32 fold), suggesting that NP-5497-KA is a more selective KOP agonist compared with nalfurafine^16–18^. NP-5497-KA also showed full agonism at the DOP and partial agonism at the MOP with relatively low potency. We preliminarily showed that NP-5497-KA is metabolically stable and rapidly absorbed with oral administration, in which the peak plasma concentration was observed 30 min after administration. Future studies of KOP biased agonism^19,20^ should be conducted, but NP-5497-KA could be a new seed compound for clinical therapy that targets the KOP.

We found that NP-5497-KA dose-dependently inhibited morphine-induced CPP. The inhibitory effect of 10 mg/kg NP-5497-KA (p.o.) on morphine-induced CPP was equivalent to 10 µg/kg nalfurafine (i.p.). These findings are consistent with previous reports that KOP agonists inhibited the rewarding effects of various addictive substances^21^. Systemic nalfurafine administration did not produce CPP or conditioned place aversion and suppressed morphine- and cocaine-induced CPP in mice and rats^5,22^. KOP agonists are a class of drugs that are gaining attention as a possible treatment for drug addiction^23,24^. Thus, NP-5497-KA, similar to other KOP agonists (e.g., nalfurafine), may be useful for the treatment of opioid and substance use disorders. KOP agonists are well known to produce aversive subjective effects, such as dysphoria^4^. In our preliminary study, conditioning with 10 mg/kg NP-5497-KA alone resulted in conditioned place aversion (data not shown). Several feasibility issues must be considered when proposing such aversive KOP agonists and other punitive agents as possible formulations for abuse deterrence along with prescription opioids. Punitive agents that reduce the nonmedical use of prescription opioids may negatively impact patient compliance if aversive effects of the punitive agent are too strong. Ideally, punitive stimuli should be employed only when a patient exceeds the prescribed dose or shortens the dosing interval. This is an issue for future studies, including measures other than those devised in the formulation. Another consideration is the complementarity of pharmacokinetic profiles of paired drugs in a formulation.

KOP agonists have been known to mediate such beneficial effects as analgesic and anti-addictive effects but have more severe adverse effects, such as anhedonia/dysphoria, sedation, anxiety, and motor incoordination. Most KOP agonists produce these untoward side effects of sufficient severity to limit their clinical utility^25^. Nalfurafine is unusual among KOP agonists. It reportedly does not produce perceptual distortions or discomfort that are typical of other KOP agonists^26^, and it has been used clinically for the management of pruritus in Japan. In the present study, both NP-5497-KA and nalfurafine significantly suppressed morphine-induced CPP but not morphine-induced hyperlocomotion, although nalfurafine tended to slightly suppress morphine-induced hyperlocomotion, similar to a previous report^22^. Furthermore, nalfurafine exerted inhibitory effects on motor incoordination in the rotarod test, whereas NP-5497-KA did not show these effects. One possible reason for these differences in effects of nalfurafine and NP-5497-KA may be differences in receptor subtype selectivity and activity. KOP agonists with a bias toward β-arrestin recruitment have a higher degree of sedation and other adverse effects than KOP agonists with a bias toward G-protein signaling^19^. One limitation of the present study is that we did not examine possible factors that may contribute to differences in adverse effects between NP-5497-KA and nalfurafine, which should be examined in future studies. Although other pharmacological effects of NP-5497-KA need to be considered, it showed potential as a seed compound for the treatment of opioid and substance use disorders with fewer adverse effects. Another limitation is that the strains of mice that were used and route of administration of the control drug nalfurafine (Supplemental Data) differed among the experiments within this study, so it will be necessary to compare data from these aligned studies in the future.

## Conclusion

We report the biological and pharmacological characterization of a novel azepane-derived ligand, NP-5497-KA, as a selective KOP agonist. In vitro studies showed that NP-5497-KA is a highly selective agonist of the KOP over the MOP and DOP. The behavioral pharmacology studies indicated that NP-5497-KA inhibited morphine-induced rewarding effects in the CPP tests in mice without causing sedation or motor incoordination in the locomotor activity and rotarod tests. NP-5497-KA could be a new seed compound for clinical therapy that targets the KOP.

### Supplementary Information

Supplementary Information.

## Data Availability

The data used in the study are available from the corresponding author on reasonable request.
