# Supplementary material for: Effects of the novel selective κ-opioid receptor agonist NP-5497-KA on morphine-induced reward-related behaviors
Source: Sci Rep. 2023 Oct 24;13:18164. doi: 10.1038/s41598-023-45584-4 (PMC10598265; doi:10.1038/s41598-023-45584-4)
Supplement: Supplementary file 1 — Supplementary Information. [file 41598_2023_45584_MOESM1_ESM.docx]

**Supplemental Methods**

*Metabolic stability in cryopreserved human and mouse hepatocytes*

The test compound was prepared to 10 mM in dimethylsulfoxide and further diluted in Krebs-Henseleit buffer (pH 7.4). Hepatocyte suspensions were prepared in Krebs-Henseleit buffer (pH 7.4) that contained 1 × 10^6^ cells/ml and a final compound concentration of 10 μM. The plate was incubated for 1 h at 37°C in a CO_2_ incubator. Samples were taken at 0, 10, 30, and 60 min and quenched with reaction-stopping solution (methanol/acetonitrile, 50/50 [v/v], containing internal standard). Samples were mixed and centrifuged at 12,000 × *g* for 10 min at 4°C, and these supernatants were applied to a high-performance liquid chromatography-ultraviolet spectroscopy system. The percentage of the remaining test compound during incubation was calculated by dividing the peak height ratio of the compound to the internal standard at a certain incubation time to that at time zero. Assays were performed on two separate occasions, each in duplicate.

*Pharmacokinetic study*

Male ICR mice received a single oral dosing of 10 mg/kg NP-5497-KA. Three animals were sacrificed at each of the selected time points (0.25, 0.5, 1, 2, and 4 h; *n* = 3/time point) after administration. Blood was collected through the abdominal vena cava under anesthesia with isoflurane, and the pooled plasma concentration of NP-5497-KA was quantified by a liquid chromatography/dual mass spectrometry system.

Table S1. Metabolic stability of NP-5497-KA in human and mouse cryopreserved hepatocytes.

|  | **Mouse** | **Human** |
| --- | --- | --- |
| *Incubation time (min)* | *Remaining ratio (%)* | |
| 0 | 100 | 100 |
| 10 | 100 | 95 |
| 30 | 95 | 92 |
| 60 | 82 | 83 |

Table S2. Concentration of 10 mg/kg NP-5497-KA in plasma after a single oral administration.

| **Time after administration (h)** | **Plasma concentration (pg/ml)** |
| --- | --- |
| 0.25 | 23,700 |
| 0.5 | 34,500 |
| 1 | 13,200 |
| 2 | 14,800 |
| 4 | 9,140 |


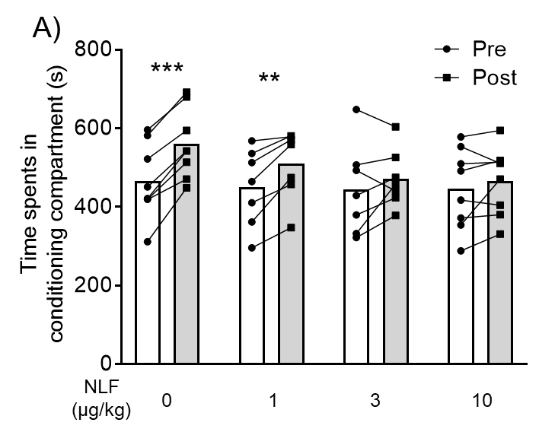

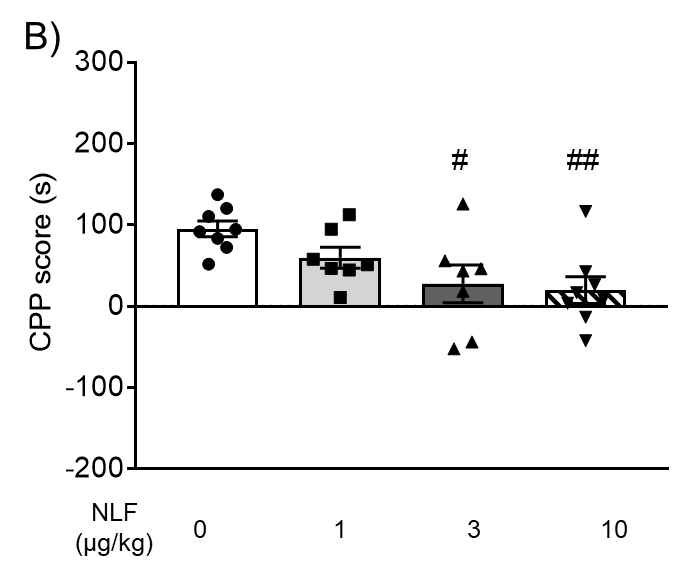


Fig. S1. Inhibitory effect of nalfurafine (NLF) on morphine-induced rewarding effects. (A) Time spent in the drug-paired compartment in the preconditioning phase (Pre, white columns) and postconditioning phase (Post, gray columns). Mice were pretreated with NLF (0, 1, 3, and 10 µg/kg, i.p.; *n* = 8, 7, 7, and 8, respectively) 5 min before each morphine treatment (10 mg/kg, i.p.) during the conditioning phase. The lines that connect symbols represent the value of each individual mouse, and the columns represent the mean. ***p* < 0.01, ****p* < 0.001, difference between pre- and postconditioning phases in each treatment. (B) Conditioned place preference (CPP) scores for each treatment in mice. The columns and vertical lines represent the mean ± SEM. ^#^*p* < 0.05, ^##^*p* < 0.01, compared with vehicle-pretreated (control) mice.


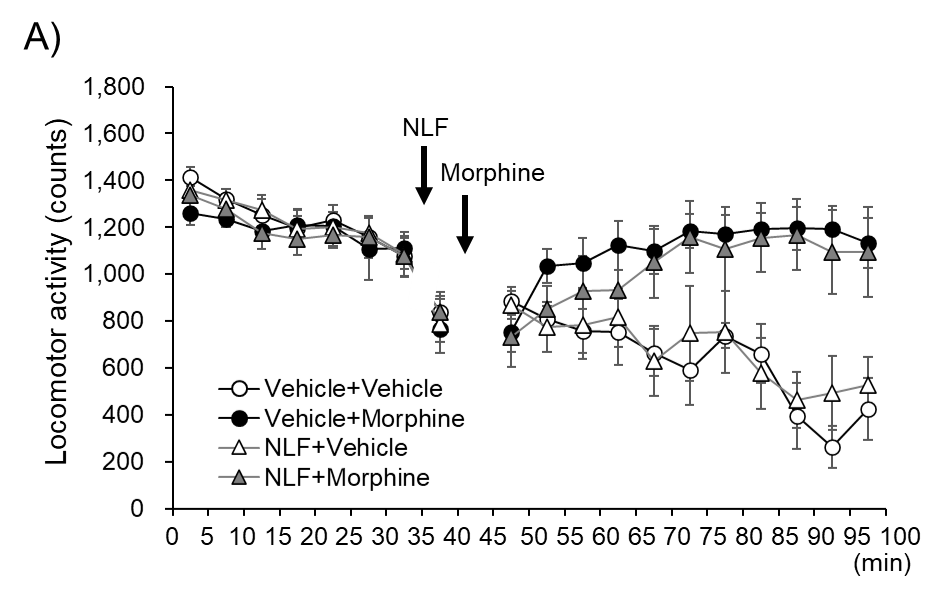

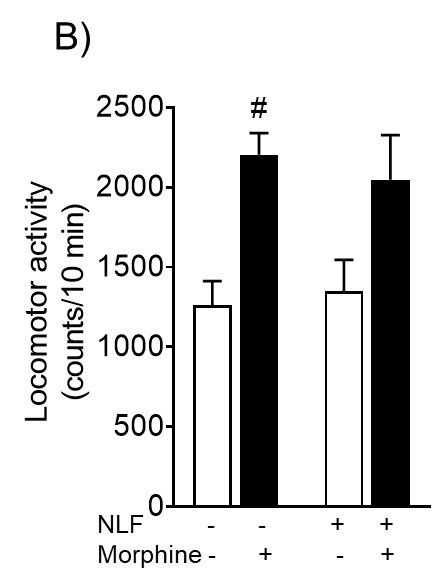


Fig. S2. Effect of nalfurafine (NLF) on morphine-induced hyperlocomotion. (A) Locomotor activity was measured before (40 min for habituation) and after (60 min) morphine treatment (10 mg/kg). (A) Mice (*n* = 6/group) were pretreated with NLF (10 µg/kg, i.p.) or vehicle (i.p.) as indicated 5 min before morphine or saline treatment (i.p.). (B) Average number of locomotor counts in 10 min blocks after morphine administration for each treatment in mice. The columns and vertical lines represent the mean ± SEM.


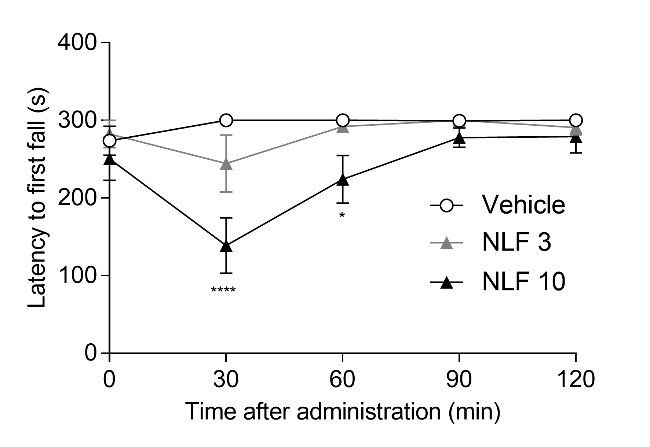


Fig. S3. Effect of nalfurafine (NLF) on rotarod performance. Rotarod performance was measured after the subcutaneous administration of NLF (3 and 10 µg/kg). Mice (*n* = 11, 10, and 11, respectively) were intraperitoneally treated with NLF (3 µg/kg: gray triangles; 10 µg/kg: black triangles) or vehicle (white circles). The data are expressed as the mean ± SEM. **p* < 0.05, *****p* < 0.0001, compared with vehicle-treated mice.
